# Does Preoperative Radio(chemo)therapy Increase Anastomotic Leakage in Rectal Cancer Surgery? A Meta-Analysis of Randomized Controlled Trials

**DOI:** 10.1155/2014/910956

**Published:** 2014-11-12

**Authors:** Changjiang Qin, Xuequn Ren, Kaiwu Xu, Zhihui Chen, Yulong He, Xinming Song

**Affiliations:** ^1^Department of Gastrointestinal Surgery, The First Affiliated Hospital of Sun Yat-sen University, Zhongshan Second Road, Guangzhou, Guangdong 510080, China; ^2^Department of Gastrointestinal Surgery, Huai He Hospital of Henan University, Kaifeng, China

## Abstract

*Objective*. Preoperative radio(chemo)therapy (pR(C)T) appears to increase postoperative complications of rectal cancer resection, but clinical trials have reported conflicting results. The objective of this meta-analysis was performed to assess the effects of pR(C)T on anastomotic leak after rectal cancer resection. *Methods*. PubMed, Embase, and the Cochrane Library were searched from January 1980 to January 2014. Randomized controlled trials included all original articles reporting anastomotic leak in patients with rectal cancer, among whom some received preoperative radiotherapy or chemoradiotherapy while others did not. The analysed end-points were the anastomotic leak. *Result*. Seven randomized controlled trials with 3375 patients were included in the meta-analysis. 1660 forming the group undergoing preoperative radiotherapy or chemoradiotherapy versus 1715 patients undergoing without preoperative radiotherapy or chemoradiotherapy. The meta-analyses found that pR(C)T was not an independent risk factor for anastomotic leakage (OR 1.02, 95% CI 0.80–1.30; *P* = 0.88). Subgroups analysis was performed and the result was not altered. *Conclusions*. Current evidence demonstrates that pR(C)T did not increase the risk of postoperative anastomotic leak after rectal cancer resection in patients.

## 1. Introduction

Current consensus in the management of locally advanced rectal cancer advocates the use of a multimodal approach to treatment. The role of preoperative radio(chemo)therapy (pR(C)T) is currently being investigated with different protocols. This approach is associated with increased downstaging and thus increased respectability [1–3]. Accordingly, pR(C)T in all UICC stages II and III rectal cancers has been recommended in the German treatment guidelines. Nevertheless, pR(C)T does not improve overall survival after rectal cancer resection and may constitute a significant overtreatment for many patients [1]. Furthermore, the concern over an increased incidence of postoperative complications has been rising [4]. Therefore, pR(C)T has been a hot issue in question in recent years.

Anastomotic leak (AL) after rectal cancer surgery is one of the most feared and potentially catastrophic early complications, with the consequent risk of immediate postoperative mortality and local recurrence [5, 6]. The reported incidence of AL varies from 1% to 30% [7–9]. Many factors have been associated with leakage of a colorectal anastomosis [8], including bowel preparation, height of the anastomosis, and level of tumor. However, it is not clear whether pR(C)T is an independent risk factor for leak following rectal resection. Some surgeons consider that pR(C)T increases the incidence of early postoperative complications, especially a higher incidence of AL [10–12]. But many large clinical experimental results have not shown increased AL rates in patients who received pR(C)T preoperatively [13–15]. In order to inform and involve patients in clinical decision-making, clinicians need reliable and accurate information on AL rates. Previous studies have shown that patients with rectal cancer prefer to be involved in the decision making process and to be informed on risks involved in the different treatment options [16].

But so far, we are unaware of any analyses in the literature summarizing the current collective data on this topic. Thus, we critically reviewed the evidence for pR(C)T on the occurrence of AL with rectal cancer. The objective of this systematic review and meta-analysis is to clarify the association between pR(C)T and anastomotic leakage and to determine the impact of pR(C)T intervention.

## 2. Materials and Methods

### 2.1. Database Search

We searched systematically the electronic databases of PubMed, Embase, and the Cochrane Library from January 1980 to January 2014. Studies that were published before 1980 were not eligible, because rectal resections were performed without standardized TME technique. The following subject headings (MeSH) terms and words were utilized: “rectal cancer,” “rectal neoplasm,” “neoadjuvant chemoradiotherapy,” “neoadjuvant radiotherapy,” “preoperative chemoradiotherapy,” “preoperative radiotherapy,” “anastomotic leak,” “anastomotic dehiscence.” Additional searches were performed by hand-searching reference lists of included studies and relevant reviews. Each study was subjected to a quality assessment by two investigators.

### 2.2. Literature Screening and Assessment

The literature screening and assessment was conducted by two professionals (QIN Chang-jiang and REN Xun-qun) with the following strict criteria.

#### 2.2.1. Inclusion Criteria

This study had to meet the following criteria and was done using the following PICOS.

1. *Population*: patients with pathologically diagnosed primary rectal cancer and with no history of preoperative radiotherapy or chemotherapy.
2. *Intervention*: anterior resection of the colorectal cancer with primary anastomosis. Patients who underwent the Hartmann procedure or abdominoperineal resection were excluded.
3. *Comparison*: patients with preoperative radiotherapy or chemoradiotherapy compared to patients without.
4. *Outcome*: AL or anastomotic dehiscence with clinical symptoms confirmed by operation or radiological diagnostic evaluation.
5. *Studies*: randomized controlled trials studies.

#### 2.2.2. Exclusion Criteria

Patients with intraperitoneal dissemination, other organ metastases, and remnant and recurrent rectal cancer were excluded. Articles that did not show AL or investigated the causes of AL were excluded. Furthermore, abstract derived from publications and chapters from books were excluded.

#### 2.2.3. Data Assessment and Quality Assessment

For the selection of studies, initially, titles and abstracts were screened. Subsequently, full-text articles were obtained to assess study eligibility. Both authors conducted the searches and identification of studies independently. When a consensus could not be reached, a third author (SONG Xin-ming) broke the tie.

The quality of randomized studies was evaluated by means of the modified Jadad score [17]. The following criteria were adopted: quality of randomization, quality of allocation concealed, quality of double-blinding, and quality of withdrawals and dropouts of the study description; each scored one point. If the method of the sequence of randomization was described by computer or randomized number, or the method of allocation concealment was described and was appropriate, or detailed description of proper methods of double blinding, an additional point was given for each item. Based on these criteria, high-quality studies scored at least four points.

### 2.3. Statistical Analysis

Meta-analyses of observational studies were performed for the outcomes of AL by the use of Review Manager 5.2 supplied by the Cochrane Collaboration. The data were estimated with the terms of AL. For dichotomous data, odds ratio (OR) was estimated with 95% confidence intervals (CI) as relevant effect measures. Heterogeneity was evaluated with the *Q*-test and the *I* ^2^ value. If *P* < 0.05 in the *Q*-test was present, significant heterogeneity was considered. Sensitivity analyses were carried out to explore reasons for heterogeneity. Furthermore, we performed a priori subgroup analyses. The Egger test was used to assess the funnel plot for significant asymmetry, indicating possible publication or other biases.

## 3. Results

### 3.1. Identification and Quality of Studies

**Figure 1:**
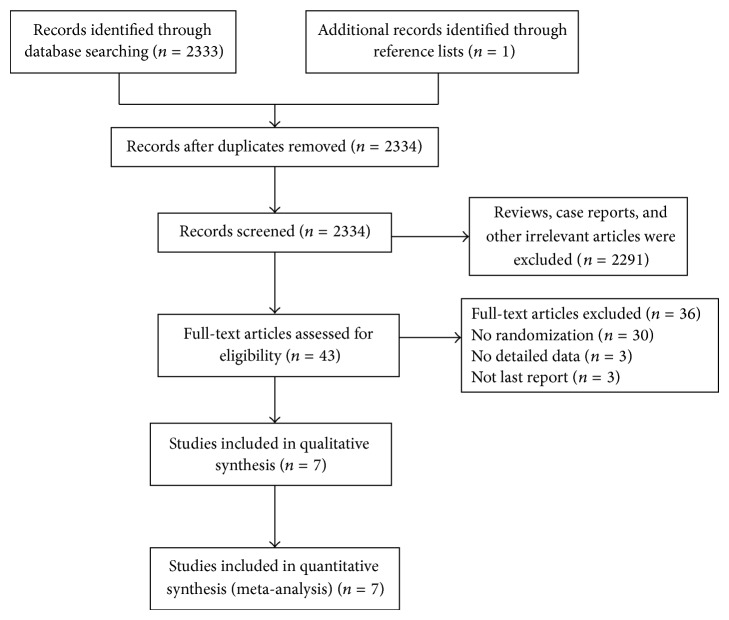
PRISMA flow diagram of the systematic article selection process.

Seven randomized controlled trials [18–24] were extracted from 2333 publications and the PRISMA flow diagram for this meta-analysis is presented in Figure 1. All seven studies stated random allocation; five [18–20, 23, 24] described the method of randomization with definite description. The concealment of allocation was made by sealed envelopes controlled by centre in two trials [22, 23]. None of the trials reported double-blind. All trials adequately presented with a detailed description of the number and the reason for patients' withdrawals and dropouts. Details of the scoring system are shown in Table 2.

### 3.2. Characteristics of Observational Studies

Seven studies published were included in the meta-analysis [18–24] and their characteristics are shown in Tables 1 and 2. All the included studies were published between 1993 and 2011, involving 3375 patients, 1660 in the pR(C)T followed by surgery group, and 1715 without neoadjuvant therapy group. Five of the studies reported on patients who received preoperative radiotherapy and two studies reported on patients who received preoperative chemoradiation.

### 3.3. Meta-Analysis of Anastomotic Leakage

**Figure 2:**
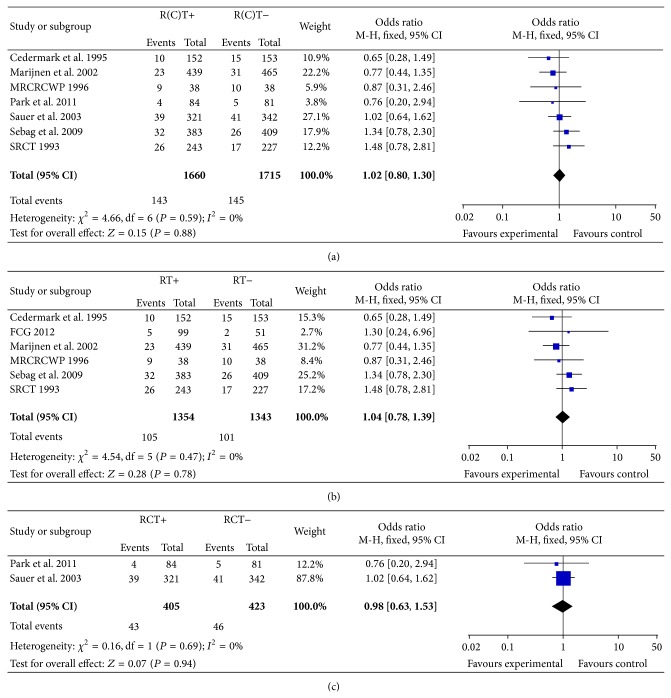
(a) Impact of pR(C)T on anastomotic leakage. CI, confidence interval; OR, odds ratio. (b) Impact of pRT on anastomotic leakage. CI, confidence interval; OR, odds ratio. (c) Impact of pRCT on anastomotic leakage. CI, confidence interval; OR, odds ratio.

We used a fixed-effects model in the meta-analysis of randomized controlled trials of pR(C)T for rectal cancer. The anastomotic leakage rate was 8.6% (143/1660) in the pR(C)T group and 8.4% (145/1715) in the without pR(C)T therapy group. The pooled OR for included trials was 1.02, with a 95% confidence interval (95% CI) of 0.81–1.30. There appeared to be no difference in the incidence of AL between two groups (*P* = 0.88) (Figure 2(a)).

Furthermore, we performed subgroup analysis by classifying the intervention arms into preoperative radiotherapy (pRT) or preoperative chemoradiotherapy (pCRT). The meta-analysis showed that PRT or PCRT plus surgery did not increase the risk of postoperative anastomotic leak to the patients with rectal cancer resection (OR = 1.04, 95% CI 0.78–1.39, *P* = 0.78 and OR = 0.98, 95% CI 0.63–1.53, *P* = 0.94), indicating the result of meta-analysis was statistically significant (Figures 2(b) and 2(c)).

### 3.4. Publication Bias

**Figure 3:**
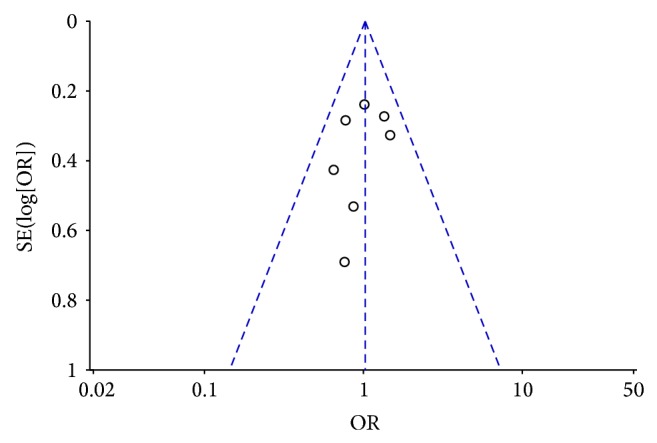
Funnel plot of the outcome of anastomotic leakage. OR, odds ratio; SE, standard error.

The funnel plot (Figure 3) of the studies included in the meta-analysis for the anastomotic leak outcome shows that none of the studies exceeded the 95% CI limit and that they were equally distributed on both sides of the vertical line. Therefore, we concluded that there was little evidence for publication bias in our study.

## 4. Discussion

Other reviews have reported that pR(C)T enhances tumor downstaging and improves local control, but they have not assessed the effects of neoadjuvant therapy on AL after rectal cancer resection [25, 26]. This is the first meta-analysis of randomized controlled trials to assess the effects of pR(C)T on AL after rectal cancer resection. Our systematic review and meta-analysis provides strong evidence that pR(C)T does not increase the incidence of rectal AL. Furthermore, there are no significant differences identified for the rates of AL complication in subgroup analysis.

The impact of anastomotic leakage on immediate postoperative mortality and local recurrence is well recognized [27, 28]. Some surgeons consider that pR(C)T increases the incidence of postoperative anastomotic leakage and they would advise the creation of a defunctioning stoma [29]. This may result in complications and further surgery is required to close it, a procedure also liable to morbidity besides the increased cost of another operation [30]. If there is no increased risk, then preventing deferral of pR(C)T (arising from potential fear of AL in locally advanced rectal cancer) may be critical for beneficial outcomes for patients [9]. Our meta-analysis showed that pR(C)T did not increase the incidence of rectal anastomotic leakage, which can help surgeons to make clinical decisions. The information of the present study may very well be used for counseling patients preoperatively.

In general, patients who receive pR(C)T tend to have more AL-associated risk factors than patients undergoing surgery alone [31–33]. Possible explanations for the increased rates of AL after preoperative chemoradiation may be due to an impaired immune system regarding both anti-infectious and antitumour immunity [34]. Furthermore, preoperative radiotherapy commonly results in local inflammation and tissue fibrosis, which could reduce wound healing and thus may increase the risk of anastomotic leakage [35].

While there is a sound scientific basis for anticipating an increased anastomotic leak rate in patients receiving pR(C)T, this finding has not been firmly established by clinical studies, with conflicting reports in the literature. In some large randomized controlled trials, Sauer and Marijnen did not observe that pR(C)T increased the risk of postoperative AL after rectal cancer resection [21, 24]. Similar findings have been reported by nonrandomized intervention studies on pooled outcome measures [13–15].

Retrospective studies have reported conflicting results as well, because retrospective design is subject to inherent biases. Martel et al. [33] performed a retrospective review of 220 cases (54 cases received pR(C)T and 166 received surgery alone) and observed no difference in clinically significant AL between the 2 groups. Garlipp et al. [31] applied propensity score to evaluate the effect of preoperative chemoradiotherapy on anastomotic leakage. They also concluded that preoperative chemoradiotherapy for rectal carcinoma did not increase the risk for AL. However, Lee et al. [32] performed a retrospective study of 1320 patients from a registry and found that pR(C)T was associated with increased risk for AL. In this study, significant imbalances between 2 treatment groups regarding the number of patients and the characteristics of the patients and their disease made it difficult to interpret the results. Likewise, Warschkow et al. [36] found that preoperative radiotherapy was associated with increased incidence of AL; they failed to find the same significance using either multivariate analysis or statistical resampling methods.

There are some limitations to the present meta-analysis that should be acknowledged. First, although seven studies were all randomized controlled trials, confounding factors such as tumor location, tumor stage, the use of diverting ileostomy, the anastomotic integrity using endoscopy or enema with contrast medium, and the definition of AL inevitably existed. Second, there was difference in the selection criteria, such as different protocols and method of procedures. Subgroup analysis according to study design was performed to address this issue, and the results indicated that the outcome was consistent across studies of different design.

Even taking into account the limitations due to the observational character of the studies included in this meta-analysis, the results indicate that pR(C)T is not an independent risk factor for postoperative AL after rectal cancer resection of patients. Our results may be useful for surgeons during patient counseling.

## Figures and Tables

**Table 1:** Baseline characteristics of studies included in the systematic review.

Study	Country	Study period	Number of patients		Treatment schedule		Anastomotic leak	
			R(C)T+	R(C)T−	R(C)T+	R(C)T−	R(C)T+	R(C)T−
Cedermark et al., 1995 [18]	Sweden	1980–1987	152	153	25 Gy, 5 fraction + S	S	10	15
MRCRCWP, 1996 [19]	England	1981–1989	38	38	40 Gy, 20 fraction + S	S	9	10
Sebag-Montefiore et al., 2009 [20]	Canada	1998–2005	383	409	25 Gy, 5 fraction + S	S + CT	32	26
Sauer et al., 2003 [21]	Germany	1995–2002	321	342	50.4 Gy, 28 fraction + 5-FU + S	S + CT	39	41
Park et al., 2011 [22]	Korea	2004–2006	84	81	46 Gy, 23 fraction + CAP + S	S + CT	4	5
Marijnen et al., 2002 [23]	Netherlands	1996–1999	439	465	25 Gy, 5 fraction + S	S	23	31
SRCT [24], 1993	Sweden	1987–1990	243	227	25.5 Gy, 5 fraction + S	S	26	17

RT = radiotherapy; CT = chemotherapy; S = surgery; 5-FU = 5-fluorouracil; Capecitabine = CAP.

**Table 2:** Jadad score of included trials.

Study	Random allocation	Concealed allocation	Double blinding	Withdrawals and dropouts	Jadad's score
Cedermark et al. [18]	2	1	0	1	4
MRCRCWP et al. [19]	2	1	0	1	4
Sebag-Montefiore et al. [20]	2	1	0	1	4
Sauer et al. [21]	1	1	0	1	3
Park et al. [22]	1	2	0	1	4
Marijnen et al. [23]	2	2	0	1	5
SRCT [24]	2	1	0	1	4
